# Bis{6-meth­oxy-2-[(4-methyl­phen­yl)­iminio­meth­yl]phenolato-κ^2^ *O*,*O*′}bis­(nitrato-κ^2^ *O*,*O*′)cadmium(II)

**DOI:** 10.1107/S1600536808033539

**Published:** 2008-10-31

**Authors:** Hua-Qiong Li, Hui-Duo Xian, Jian-Feng Liu, Guo-Liang Zhao

**Affiliations:** a Zhejiang Key Laboratory for Reactive Chemistry on Solid Surfaces, Institute of Physical Chemistry, Zhejiang Normal University, Jinhua, Zhejiang 321004, People’s Republic of China, and, College of Chemistry and Life Science, Zhejiang Normal University, Jinhua 321004, Zhejiang, People’s Republic of China

## Abstract

The Schiff base 6-meth­oxy-2-[(4-methyl­phen­yl)imino­meth­yl]­phenol (HL) forms a neutral complex with cadmium(II) nitrate, [Cd(NO_3_)_2_(C_15_H_15_NO_2_)_2_], in which the four O atoms of the two independent ligands are coordinated to the metal center and the protonated imine N atoms are involved in a hydrogen bond with the phenoxide group. Intra­molecular N—H⋯O hydrogen-bonding inter­actions stabilize the structure. Each organic ligand assumes a zwitterionic form, chelating to the metal atom through the two O atoms, while the two nitrate groups also exhibit chelating behavior, leading to a distorted octahedral coordination of the Cd atom.

## Related literature

For related literature, see: Dominiak *et al.* (2003 ▶); Elmali *et al.* (2003 ▶); Filarowski *et al.* (1998 ▶); Müller *et al.* (2001 ▶); Novitchi *et al.* (2008 ▶); Schiff (1864 ▶); West (1960 ▶); Woźniak *et al.* (1995 ▶); Yu *et al.* (2007 ▶); Zhao *et al.* (2007 ▶); Zhou *et al.* (2007 ▶); Zhou & Zhao (2007 ▶).
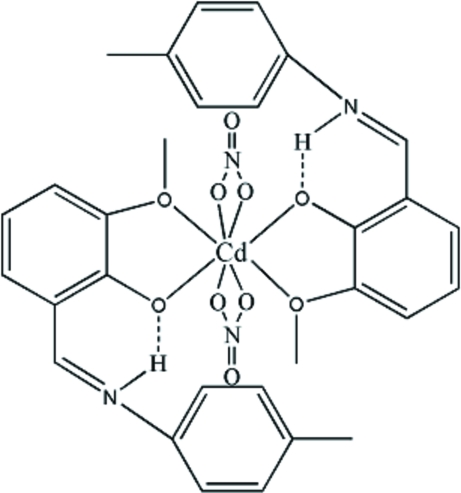


## Experimental

### 

#### Crystal data

[Cd(NO_3_)_2_(C_15_H_15_NO_2_)_2_]
*M* *_r_* = 718.99
Monoclinic, 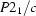

*a* = 10.8009 (4) Å
*b* = 27.3377 (10) Å
*c* = 10.5878 (4) Å
β = 90.208 (2)°
*V* = 3126.3 (2) Å^3^
*Z* = 4
Mo *K*α radiation
μ = 0.76 mm^−1^
*T* = 296 (2) K
0.35 × 0.30 × 0.11 mm

#### Data collection

Bruker APEXII area-detector diffractometer
Absorption correction: multi-scan (*SADABS*; Sheldrick, 1996 ▶) *T*_min_ = 0.765, *T*_max_ = 0.923
31192 measured reflections
5498 independent reflections
3112 reflections with *I* > 2σ(*I*)
*R*_int_ = 0.090

#### Refinement

*R*[*F* ^2^ > 2σ(*F* ^2^)] = 0.059
*wR*(*F* ^2^) = 0.178
*S* = 0.98
5498 reflections
389 parameters
3 restraints
H-atom parameters constrained
Δρ_max_ = 0.92 e Å^−3^
Δρ_min_ = −0.75 e Å^−3^

### 

Data collection: *APEX2* (Bruker, 2006 ▶); cell refinement: *SAINT* (Bruker, 2006 ▶); data reduction: *SAINT*; program(s) used to solve structure: *SHELXTL* (Sheldrick, 2008 ▶); program(s) used to refine structure: *SHELXTL*; molecular graphics: *SHELXTL*; software used to prepare material for publication: *SHELXTL*.

## Supplementary Material

Crystal structure: contains datablocks I, global. DOI: 10.1107/S1600536808033539/at2640sup1.cif

Structure factors: contains datablocks I. DOI: 10.1107/S1600536808033539/at2640Isup2.hkl

Additional supplementary materials:  crystallographic information; 3D view; checkCIF report

## Figures and Tables

**Table 1:** Hydrogen-bond geometry (Å, °)

*D*—H⋯*A*	*D*—H	H⋯*A*	*D*⋯*A*	*D*—H⋯*A*
N1—H1*D*⋯O2	0.86	1.94	2.616 (6)	135
N2—H2*A*⋯O4	0.86	1.90	2.577 (7)	135

**Fig. 1.**
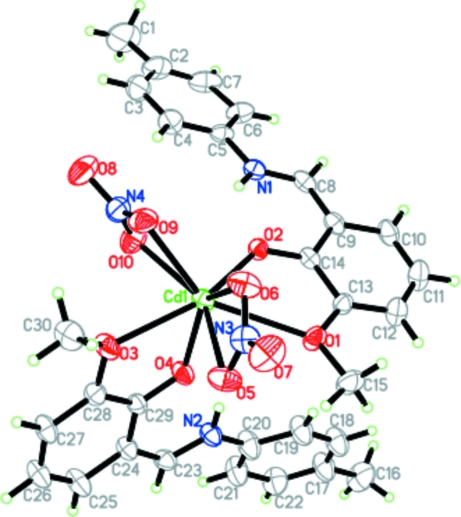
The molecular structure of complex, showing the atom-labelling scheme. Displacement ellipsoids are drawn at the 30% probability level.

**Fig. 2.**
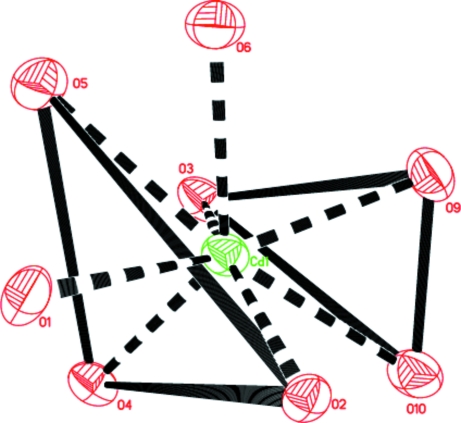
Eight-coordinate geometry of Cd.

## supplementary crystallographic information

### Comment

Since the Schiff bases, the products of condensation of carbonyl compounds with primary amines, were discovered in 1864 by Hugo Schiff (Schiff, 1864), the studies on this kind of compounds containing imine group were carried out widely in different application fields. And recently, the metal complexes with the Schiff base ligands derived from substituted salicylaldehyde and aniline, have received attention due to their applications in catalysis, nuclear medicine (Zhou *et al.*, 2007), magnetism (Elmali *et al.*, 2003) and novel structural features (Müller *et al.*, 2001; Novitchi *et al.*, 2008). They include complexes with a methoxy group in the *ortho* position (West, 1960) which can bind to the metal too. Zhao and co-workers had reported complexes of this series with several transitional and rare earth metals (Zhou & Zhao, 2007; Yu *et al.*, 2007; Zhao *et al.*, 2007). Here we decribe the synthesis and crystal structure of a new cadmium(II) complex (Fig. 1), Cd(HL)_2_(NO_3_)_2_, involving the Schiff base HL.

The most interesting feature of the complex is the two N—H···O intramolecular hydrogen bonds. In fact, there is a proton-transfer equilibrium between the OH and NH tautomers (Dominiak *et al.*, 2003). And it is reported that the products of condensation of salicylaldehydes with anilines show intermolecular proton-transfer equilibrium and double fluorescence (Filarowski *et al.*, 1998; Woźniak *et al.*, 1995). In addition, the title complex has the Cd atom in a geometry that can be better described as a bicapped trigonal antiprism (Fig. 2).

### Experimental

First, the ligand was prepared by the direct solid-phase reaction of *o*-vanillin (10 mmol, 1.5251 g) and *p*-toluidine (10 mmol, 1.0700 g). The reactants were ground in an agate mortar. The colour of the mixture changed from light yellow to orange. Then, for the preparation of the complex, the solution of Cd(NO_3_)_2_.4H_2_O (1 mmol, 0.3091 g) in methanol (10 ml) was added to a methanol (30 ml) solution of the Schiff base ligand (2 mmol, 0.4812 g). Red crystals were obtained after two weeks.

### Refinement

The H atoms bonded to C and N atoms were positioned geometrically and refined using a riding model [aromatic C—H = 0.93 Å, methylic C—H = 0.96 Å, N—H = 0.86 Å, *U*_iso_(H) = 1.2 or 1.5*U*_eq_(C)].

### Crystal data

**Table:**

[Cd(NO_3_)_2_(C_15_H_15_NO_2_)_2_]	*F*(000) = 1464
*M**_r_* = 718.99	*D*_x_ = 1.528 Mg m^−^^3^
Monoclinic, *P*2_1_/*c*	Mo *K*α radiation, λ = 0.71073 Å
Hall symbol: -P 2ybc	Cell parameters from 8740 reflections
*a* = 10.8009 (4) Å	θ = 1.9–25.0°
*b* = 27.3377 (10) Å	µ = 0.76 mm^−^^1^
*c* = 10.5878 (4) Å	*T* = 296 K
β = 90.208 (2)°	Block, red
*V* = 3126.3 (2) Å^3^	0.35 × 0.30 × 0.11 mm
*Z* = 4	

### Data collection

**Table:**

Bruker APEXII area-detector diffractometer	5498 independent reflections
Radiation source: fine-focus sealed tube	3112 reflections with *I* > 2σ(*I*)
graphite	*R*_int_ = 0.090
ω scans	θ_max_ = 25.0°, θ_min_ = 1.9°
Absorption correction: multi-scan (SADABS; Sheldrick, 1996)	*h* = −12→12
*T*_min_ = 0.765, *T*_max_ = 0.923	*k* = −32→32
31192 measured reflections	*l* = −12→12

### Refinement

**Table:**

Refinement on *F*^2^	Secondary atom site location: difference Fourier map
Least-squares matrix: full	Hydrogen site location: inferred from neighbouring sites
*R*[*F*^2^ > 2σ(*F*^2^)] = 0.059	H-atom parameters constrained
*wR*(*F*^2^) = 0.178	*w* = 1/[σ^2^(*F*_o_^2^) + (0.1*P*)^2^] where *P* = (*F*_o_^2^ + 2*F*_c_^2^)/3
*S* = 0.98	(Δ/σ)_max_ = 0.003
5498 reflections	Δρ_max_ = 0.92 e Å^−^^3^
389 parameters	Δρ_min_ = −0.74 e Å^−^^3^
3 restraints	Extinction correction: SHELXTL (Sheldrick, 2008), Fc^*^=kFc[1+0.001xFc^2^λ^3^/sin(2θ)]^-1/4^
Primary atom site location: structure-invariant direct methods	Extinction coefficient: 0.0013 (4)

### Special details

**Table:**

Geometry. All e.s.d.'s (except the e.s.d. in the dihedral angle between two l.s. planes) are estimated using the full covariance matrix. The cell e.s.d.'s are taken into account individually in the estimation of e.s.d.'s in distances, angles and torsion angles; correlations between e.s.d.'s in cell parameters are only used when they are defined by crystal symmetry. An approximate (isotropic) treatment of cell e.s.d.'s is used for estimating e.s.d.'s involving l.s. planes.
Refinement. Refinement of *F*^2^ against ALL reflections. The weighted *R*-factor *wR* and goodness of fit *S* are based on *F*^2^, conventional *R*-factors *R* are based on *F*, with *F* set to zero for negative *F*^2^. The threshold expression of *F*^2^ > σ(*F*^2^) is used only for calculating *R*-factors(gt) *etc*. and is not relevant to the choice of reflections for refinement. *R*-factors based on *F*^2^ are statistically about twice as large as those based on *F*, and *R*- factors based on ALL data will be even larger.

### Fractional atomic coordinates and isotropic or equivalent isotropic displacement parameters (Å^2^)

**Table:**

	*x*	*y*	*z*	*U*_iso_*/*U*_eq_	
Cd1	0.52395 (5)	0.120056 (16)	0.72358 (4)	0.0622 (2)	
N1	0.6050 (5)	0.0522 (2)	0.3386 (4)	0.0599 (14)	
H1D	0.6111	0.0635	0.4141	0.072*	
O1	0.2880 (4)	0.08774 (19)	0.6790 (4)	0.0758 (14)	
C1	1.0356 (10)	0.0759 (4)	0.0381 (11)	0.177 (6)	
H1A	1.1044	0.0861	0.0893	0.265*	
H1B	1.0161	0.1011	−0.0220	0.265*	
H1C	1.0567	0.0464	−0.0060	0.265*	
O2	0.4950 (4)	0.07844 (15)	0.5476 (3)	0.0573 (11)	
N2	0.2895 (5)	0.2425 (2)	0.5429 (5)	0.0717 (16)	
H2A	0.3209	0.2145	0.5612	0.086*	
C2	0.9263 (10)	0.0668 (4)	0.1200 (10)	0.110 (3)	
O3	0.6180 (5)	0.18717 (18)	0.8523 (4)	0.0833 (16)	
N3	0.4284 (6)	0.0859 (3)	0.9582 (6)	0.0827 (19)	
C3	0.9270 (9)	0.0774 (3)	0.2471 (10)	0.110 (3)	
H3A	0.9996	0.0881	0.2857	0.132*	
O4	0.4455 (4)	0.19201 (16)	0.6736 (4)	0.0681 (12)	
N4	0.7851 (7)	0.1171 (3)	0.6646 (8)	0.088 (2)	
C4	0.8199 (8)	0.0723 (3)	0.3176 (7)	0.090 (2)	
H4A	0.8209	0.0800	0.4032	0.108*	
O5	0.4001 (6)	0.1286 (2)	0.9256 (5)	0.0974 (18)	
C5	0.7126 (7)	0.0561 (2)	0.2624 (6)	0.0632 (17)	
O6	0.4965 (5)	0.06211 (19)	0.8850 (5)	0.0908 (16)	
C6	0.7084 (7)	0.0448 (3)	0.1345 (6)	0.085 (2)	
H6A	0.6371	0.0326	0.0963	0.102*	
O7	0.3892 (6)	0.0678 (2)	1.0554 (5)	0.118 (2)	
C7	0.8180 (10)	0.0526 (4)	0.0660 (8)	0.107 (3)	
H7A	0.8161	0.0478	−0.0209	0.129*	
C8	0.4994 (7)	0.0337 (2)	0.3078 (6)	0.0651 (19)	
H8A	0.4935	0.0178	0.2303	0.078*	
O8	0.8962 (7)	0.1137 (3)	0.6465 (8)	0.148 (3)	
O9	0.7315 (5)	0.0961 (2)	0.7569 (5)	0.0932 (15)	
C9	0.3925 (7)	0.0357 (2)	0.3828 (6)	0.0635 (18)	
O10	0.7174 (5)	0.1395 (2)	0.5923 (5)	0.0983 (17)	
C10	0.2830 (8)	0.0150 (3)	0.3372 (7)	0.082 (2)	
H10A	0.2832	−0.0015	0.2604	0.098*	
C11	0.1761 (9)	0.0187 (3)	0.4042 (8)	0.103 (3)	
H11A	0.1039	0.0046	0.3730	0.124*	
C12	0.1741 (8)	0.0431 (3)	0.5180 (7)	0.089 (2)	
H12A	0.0999	0.0459	0.5617	0.107*	
C13	0.2790 (6)	0.0631 (2)	0.5672 (6)	0.0599 (17)	
C14	0.3929 (6)	0.0600 (2)	0.5020 (5)	0.0560 (16)	
C15	0.1807 (7)	0.0849 (3)	0.7610 (7)	0.098 (3)	
H15B	0.1649	0.0514	0.7821	0.147*	
H15C	0.1100	0.0984	0.7183	0.147*	
H30A	0.1965	0.1032	0.8369	0.147*	
C16	−0.1017 (8)	0.2383 (4)	0.1810 (8)	0.135 (4)	
H16A	−0.1112	0.2701	0.1441	0.203*	
H16B	−0.1776	0.2288	0.2210	0.203*	
H16C	−0.0817	0.2151	0.1161	0.203*	
C17	0.0001 (9)	0.2395 (3)	0.2769 (9)	0.1052 (15)	
C18	0.0332 (8)	0.2003 (3)	0.3448 (8)	0.1052 (15)	
H18A	−0.0100	0.1714	0.3320	0.126*	
C19	0.1278 (9)	0.2004 (3)	0.4330 (8)	0.1052 (15)	
H19A	0.1452	0.1725	0.4799	0.126*	
C20	0.1959 (9)	0.2424 (3)	0.4501 (9)	0.1052 (15)	
C21	0.1634 (8)	0.2822 (3)	0.3850 (8)	0.105 (3)	
H21A	0.2063	0.3114	0.3967	0.126*	
C22	0.0648 (8)	0.2800 (3)	0.2990 (8)	0.105 (3)	
H22A	0.0434	0.3083	0.2553	0.126*	
C23	0.3343 (7)	0.2797 (3)	0.6043 (6)	0.0704 (19)	
H23A	0.3031	0.3105	0.5846	0.084*	
C24	0.4257 (7)	0.2769 (2)	0.6977 (6)	0.0648 (18)	
C25	0.4601 (8)	0.3203 (3)	0.7604 (7)	0.091 (3)	
H25A	0.4252	0.3500	0.7369	0.109*	
C26	0.5450 (8)	0.3182 (3)	0.8558 (7)	0.092 (3)	
H26A	0.5659	0.3467	0.8988	0.111*	
C27	0.6008 (8)	0.2744 (3)	0.8898 (6)	0.081 (2)	
H27A	0.6598	0.2737	0.9540	0.097*	
C28	0.5685 (7)	0.2320 (3)	0.8280 (6)	0.0646 (18)	
C29	0.4791 (6)	0.2322 (2)	0.7297 (6)	0.0576 (16)	
C30	0.6976 (8)	0.1818 (3)	0.9596 (7)	0.105 (3)	
H30B	0.7201	0.2136	0.9910	0.158*	
H30C	0.7709	0.1644	0.9355	0.158*	
H30D	0.6551	0.1640	1.0244	0.158*	

### Atomic displacement parameters (Å^2^)

**Table:**

	*U*^11^	*U*^22^	*U*^33^	*U*^12^	*U*^13^	*U*^23^
Cd1	0.0739 (4)	0.0603 (4)	0.0524 (3)	0.0070 (3)	−0.0105 (2)	−0.0061 (2)
N1	0.074 (4)	0.060 (3)	0.047 (3)	0.005 (3)	0.000 (3)	−0.003 (2)
O1	0.057 (3)	0.108 (4)	0.062 (3)	−0.009 (3)	0.013 (2)	−0.016 (3)
C1	0.154 (11)	0.172 (12)	0.206 (12)	−0.010 (9)	0.122 (10)	0.007 (10)
O2	0.057 (3)	0.067 (3)	0.048 (2)	−0.005 (2)	−0.003 (2)	−0.012 (2)
N2	0.087 (4)	0.056 (4)	0.072 (4)	0.002 (3)	−0.016 (3)	0.000 (3)
C2	0.114 (8)	0.111 (8)	0.106 (8)	0.015 (6)	0.059 (7)	0.008 (6)
O3	0.099 (4)	0.074 (4)	0.076 (3)	0.013 (3)	−0.043 (3)	−0.019 (3)
N3	0.093 (5)	0.099 (6)	0.055 (4)	0.011 (4)	−0.013 (3)	0.002 (4)
C3	0.101 (7)	0.096 (7)	0.134 (8)	−0.018 (5)	0.038 (6)	−0.008 (6)
O4	0.080 (3)	0.056 (3)	0.069 (3)	0.002 (2)	−0.022 (2)	−0.009 (2)
N4	0.075 (5)	0.099 (6)	0.090 (5)	−0.002 (4)	0.001 (4)	−0.023 (4)
C4	0.099 (7)	0.096 (6)	0.075 (5)	−0.008 (5)	0.026 (5)	−0.006 (4)
O5	0.128 (5)	0.097 (5)	0.068 (3)	0.024 (4)	0.014 (2)	−0.012 (3)
C5	0.073 (5)	0.060 (4)	0.056 (4)	0.014 (4)	0.019 (3)	0.004 (3)
O6	0.126 (5)	0.078 (3)	0.069 (3)	0.021 (3)	0.010 (3)	0.0088 (19)
C6	0.091 (6)	0.108 (6)	0.054 (4)	0.028 (5)	0.008 (4)	0.001 (4)
O7	0.120 (5)	0.160 (6)	0.074 (4)	−0.002 (4)	0.021 (3)	0.033 (4)
C7	0.138 (9)	0.119 (8)	0.065 (5)	0.046 (7)	0.041 (6)	0.012 (5)
C8	0.102 (6)	0.053 (4)	0.041 (3)	0.000 (4)	−0.003 (4)	−0.004 (3)
O8	0.069 (5)	0.185 (8)	0.190 (8)	0.017 (4)	−0.015 (5)	−0.039 (5)
O9	0.086 (2)	0.099 (4)	0.095 (4)	0.027 (3)	−0.017 (3)	−0.006 (3)
C9	0.088 (5)	0.053 (4)	0.049 (4)	−0.006 (4)	−0.002 (3)	−0.004 (3)
O10	0.074 (4)	0.129 (5)	0.092 (4)	0.000 (4)	−0.020 (3)	0.018 (4)
C10	0.097 (7)	0.078 (5)	0.070 (5)	−0.020 (5)	−0.010 (4)	−0.020 (4)
C11	0.092 (7)	0.118 (8)	0.100 (7)	−0.039 (6)	−0.005 (5)	−0.007 (6)
C12	0.079 (6)	0.103 (7)	0.084 (5)	−0.025 (5)	−0.003 (4)	−0.001 (5)
C13	0.055 (4)	0.067 (5)	0.058 (4)	−0.004 (3)	0.005 (3)	−0.002 (3)
C14	0.076 (5)	0.047 (4)	0.045 (3)	−0.003 (3)	−0.004 (3)	0.001 (3)
C15	0.076 (6)	0.126 (8)	0.091 (6)	0.003 (5)	0.028 (4)	−0.021 (5)
C16	0.098 (8)	0.212 (13)	0.097 (7)	0.001 (7)	−0.038 (6)	−0.023 (7)
C17	0.121 (4)	0.090 (4)	0.104 (3)	−0.013 (3)	−0.045 (3)	−0.003 (3)
C18	0.121 (4)	0.090 (4)	0.104 (3)	−0.013 (3)	−0.045 (3)	−0.003 (3)
C19	0.121 (4)	0.090 (4)	0.104 (3)	−0.013 (3)	−0.045 (3)	−0.003 (3)
C20	0.121 (4)	0.090 (4)	0.104 (3)	−0.013 (3)	−0.045 (3)	−0.003 (3)
C21	0.113 (7)	0.086 (6)	0.116 (7)	−0.010 (5)	−0.053 (6)	0.017 (5)
C22	0.098 (7)	0.109 (8)	0.108 (7)	0.006 (5)	−0.041 (5)	0.019 (5)
C23	0.078 (5)	0.057 (5)	0.076 (5)	0.012 (4)	−0.006 (4)	0.001 (4)
C24	0.082 (5)	0.060 (4)	0.053 (4)	−0.001 (4)	−0.015 (4)	0.000 (3)
C25	0.131 (8)	0.058 (5)	0.083 (5)	−0.003 (4)	−0.031 (5)	−0.005 (4)
C26	0.122 (8)	0.066 (5)	0.089 (6)	−0.011 (5)	−0.020 (5)	−0.018 (4)
C27	0.107 (7)	0.079 (6)	0.056 (4)	−0.015 (5)	−0.021 (4)	−0.012 (4)
C28	0.069 (5)	0.074 (5)	0.051 (4)	−0.001 (4)	−0.004 (3)	−0.008 (3)
C29	0.060 (4)	0.057 (4)	0.056 (4)	−0.001 (3)	−0.003 (3)	−0.006 (3)
C30	0.125 (8)	0.107 (7)	0.083 (5)	0.019 (6)	−0.048 (5)	−0.013 (5)

### Geometric parameters (Å, °)

**Table:**

Cd1—O2	2.204 (4)	C9—C10	1.397 (9)
Cd1—O4	2.205 (4)	C9—C14	1.426 (8)
Cd1—O6	2.350 (5)	C10—C11	1.361 (11)
Cd1—O9	2.361 (5)	C10—H10A	0.9300
Cd1—O3	2.500 (5)	C11—C12	1.377 (11)
Cd1—O5	2.538 (5)	C11—H11A	0.9300
Cd1—O10	2.569 (6)	C12—C13	1.360 (9)
N1—C8	1.288 (8)	C12—H12A	0.9300
N1—C5	1.421 (8)	C13—C14	1.416 (9)
N1—H1D	0.8600	C15—H15B	0.9600
O1—C13	1.366 (7)	C15—H15C	0.9600
O1—C15	1.452 (7)	C15—H30A	0.9600
C1—C2	1.488 (11)	C16—C17	1.495 (11)
C1—H1A	0.9600	C16—H16A	0.9600
C1—H1B	0.9600	C16—H16B	0.9600
C1—H1C	0.9600	C16—H16C	0.9600
O2—C14	1.304 (7)	C17—C22	1.330 (11)
N2—C23	1.299 (8)	C17—C18	1.337 (12)
N2—C20	1.407 (9)	C18—C19	1.381 (11)
N2—H2A	0.8600	C18—H18A	0.9300
C2—C7	1.356 (12)	C19—C20	1.376 (12)
C2—C3	1.376 (12)	C19—H19A	0.9300
O3—C28	1.361 (8)	C20—C21	1.334 (11)
O3—C30	1.429 (8)	C21—C22	1.400 (10)
N3—O7	1.220 (7)	C21—H21A	0.9300
N3—O6	1.253 (7)	C22—H22A	0.9300
N3—O5	1.254 (8)	C23—C24	1.397 (9)
C3—C4	1.386 (10)	C23—H23A	0.9300
C3—H3A	0.9300	C24—C29	1.394 (9)
O4—C29	1.299 (7)	C24—C25	1.407 (9)
N4—O8	1.219 (9)	C25—C26	1.362 (10)
N4—O10	1.222 (9)	C25—H25A	0.9300
N4—O9	1.274 (8)	C26—C27	1.390 (10)
C4—C5	1.371 (10)	C26—H26A	0.9300
C4—H4A	0.9300	C27—C28	1.374 (9)
C5—C6	1.389 (9)	C27—H27A	0.9300
C6—C7	1.407 (11)	C28—C29	1.418 (9)
C6—H6A	0.9300	C30—H30B	0.9600
C7—H7A	0.9300	C30—H30C	0.9600
C8—C9	1.404 (9)	C30—H30D	0.9600
C8—H8A	0.9300		
			
O2—Cd1—O4	101.81 (15)	C11—C10—C9	120.6 (7)
O2—Cd1—O6	104.42 (17)	C11—C10—H10A	119.7
O4—Cd1—O6	136.58 (18)	C9—C10—H10A	119.7
O2—Cd1—O9	96.59 (17)	C10—C11—C12	120.6 (8)
O4—Cd1—O9	130.3 (2)	C10—C11—H11A	119.7
O6—Cd1—O9	80.0 (2)	C12—C11—H11A	119.7
O2—Cd1—O3	153.63 (17)	C13—C12—C11	120.9 (8)
O4—Cd1—O3	68.35 (15)	C13—C12—H12A	119.5
O6—Cd1—O3	98.63 (18)	C11—C12—H12A	119.5
O9—Cd1—O3	74.81 (18)	C12—C13—O1	126.0 (6)
O2—Cd1—O5	133.35 (18)	C12—C13—C14	120.9 (6)
O4—Cd1—O5	85.23 (18)	O1—C13—C14	113.1 (5)
O6—Cd1—O5	51.73 (17)	O2—C14—C13	122.1 (5)
O9—Cd1—O5	113.7 (2)	O2—C14—C9	120.5 (6)
O3—Cd1—O5	71.77 (19)	C13—C14—C9	117.3 (6)
O2—Cd1—O10	76.28 (18)	O1—C15—H15B	109.5
O4—Cd1—O10	89.88 (18)	O1—C15—H15C	109.5
O6—Cd1—O10	129.65 (19)	H15B—C15—H15C	109.5
O9—Cd1—O10	50.56 (19)	O1—C15—H30A	109.5
O3—Cd1—O10	79.22 (19)	H15B—C15—H30A	109.5
O5—Cd1—O10	150.3 (2)	H15C—C15—H30A	109.5
C8—N1—C5	127.6 (6)	C17—C16—H16A	109.5
C8—N1—H1D	116.2	C17—C16—H16B	109.5
C5—N1—H1D	116.2	H16A—C16—H16B	109.5
C13—O1—C15	116.0 (5)	C17—C16—H16C	109.5
C2—C1—H1A	109.5	H16A—C16—H16C	109.5
C2—C1—H1B	109.5	H16B—C16—H16C	109.5
H1A—C1—H1B	109.5	C22—C17—C18	115.6 (8)
C2—C1—H1C	109.5	C22—C17—C16	121.5 (9)
H1A—C1—H1C	109.5	C18—C17—C16	122.9 (9)
H1B—C1—H1C	109.5	C17—C18—C19	123.9 (9)
C14—O2—Cd1	129.0 (4)	C17—C18—H18A	118.0
C23—N2—C20	128.0 (7)	C19—C18—H18A	118.0
C23—N2—H2A	116.0	C20—C19—C18	118.9 (9)
C20—N2—H2A	116.0	C20—C19—H19A	120.5
C7—C2—C3	118.3 (8)	C18—C19—H19A	120.5
C7—C2—C1	119.1 (10)	C21—C20—C19	118.2 (9)
C3—C2—C1	122.2 (11)	C21—C20—N2	123.2 (8)
C28—O3—C30	118.5 (6)	C19—C20—N2	118.4 (8)
C28—O3—Cd1	113.5 (4)	C20—C21—C22	120.0 (9)
C30—O3—Cd1	126.9 (5)	C20—C21—H21A	120.0
O7—N3—O6	121.2 (8)	C22—C21—H21A	120.0
O7—N3—O5	121.6 (7)	C17—C22—C21	123.2 (9)
O6—N3—O5	117.2 (6)	C17—C22—H22A	118.4
C2—C3—C4	120.3 (9)	C21—C22—H22A	118.4
C2—C3—H3A	119.9	N2—C23—C24	125.0 (7)
C4—C3—H3A	119.9	N2—C23—H23A	117.5
C29—O4—Cd1	122.5 (4)	C24—C23—H23A	117.5
O8—N4—O10	121.9 (8)	C29—C24—C23	120.6 (6)
O8—N4—O9	122.4 (9)	C29—C24—C25	121.1 (6)
O10—N4—O9	115.7 (7)	C23—C24—C25	118.3 (7)
C5—C4—C3	120.6 (8)	C26—C25—C24	119.4 (7)
C5—C4—H4A	119.7	C26—C25—H25A	120.3
C3—C4—H4A	119.7	C24—C25—H25A	120.3
N3—O5—Cd1	91.0 (4)	C25—C26—C27	121.2 (7)
C4—C5—C6	120.8 (7)	C25—C26—H26A	119.4
C4—C5—N1	118.3 (6)	C27—C26—H26A	119.4
C6—C5—N1	120.9 (7)	C28—C27—C26	119.7 (7)
N3—O6—Cd1	100.1 (4)	C28—C27—H27A	120.2
C5—C6—C7	116.4 (8)	C26—C27—H27A	120.2
C5—C6—H6A	121.8	O3—C28—C27	124.7 (7)
C7—C6—H6A	121.8	O3—C28—C29	114.1 (6)
C2—C7—C6	123.5 (8)	C27—C28—C29	121.2 (7)
C2—C7—H7A	118.3	O4—C29—C24	121.1 (6)
C6—C7—H7A	118.3	O4—C29—C28	121.4 (6)
N1—C8—C9	124.7 (6)	C24—C29—C28	117.5 (6)
N1—C8—H8A	117.6	O3—C30—H30B	109.5
C9—C8—H8A	117.6	O3—C30—H30C	109.5
N4—O9—Cd1	101.2 (5)	H30B—C30—H30C	109.5
C10—C9—C8	119.0 (6)	O3—C30—H30D	109.5
C10—C9—C14	119.7 (7)	H30B—C30—H30D	109.5
C8—C9—C14	121.3 (6)	H30C—C30—H30D	109.5
N4—O10—Cd1	92.5 (5)		
			
O4—Cd1—O2—C14	−75.0 (5)	N1—C8—C9—C14	0.9 (11)
O6—Cd1—O2—C14	70.1 (5)	O8—N4—O10—Cd1	179.5 (7)
O9—Cd1—O2—C14	151.4 (5)	O9—N4—O10—Cd1	2.0 (7)
O3—Cd1—O2—C14	−139.7 (5)	O2—Cd1—O10—N4	−112.0 (5)
O5—Cd1—O2—C14	19.6 (6)	O4—Cd1—O10—N4	145.9 (5)
O10—Cd1—O2—C14	−161.9 (5)	O6—Cd1—O10—N4	−14.4 (6)
O2—Cd1—O3—C28	74.8 (6)	O9—Cd1—O10—N4	−1.2 (4)
O4—Cd1—O3—C28	2.5 (4)	O3—Cd1—O10—N4	77.9 (5)
O6—Cd1—O3—C28	−134.4 (5)	O5—Cd1—O10—N4	65.7 (7)
O9—Cd1—O3—C28	148.5 (5)	C8—C9—C10—C11	−176.4 (7)
O5—Cd1—O3—C28	−89.6 (5)	C14—C9—C10—C11	1.5 (11)
O10—Cd1—O3—C28	96.7 (5)	C9—C10—C11—C12	0.2 (14)
O2—Cd1—O3—C30	−117.5 (6)	C10—C11—C12—C13	−1.5 (14)
O4—Cd1—O3—C30	170.3 (7)	C11—C12—C13—O1	−179.6 (7)
O6—Cd1—O3—C30	33.4 (6)	C11—C12—C13—C14	1.0 (12)
O9—Cd1—O3—C30	−43.7 (6)	C15—O1—C13—C12	11.0 (10)
O5—Cd1—O3—C30	78.2 (6)	C15—O1—C13—C14	−169.6 (6)
O10—Cd1—O3—C30	−95.5 (6)	Cd1—O2—C14—C13	−2.6 (8)
C7—C2—C3—C4	1.7 (15)	Cd1—O2—C14—C9	177.4 (4)
C1—C2—C3—C4	174.4 (9)	C12—C13—C14—O2	−179.3 (6)
O2—Cd1—O4—C29	−155.5 (5)	O1—C13—C14—O2	1.2 (9)
O6—Cd1—O4—C29	78.3 (5)	C12—C13—C14—C9	0.7 (10)
O9—Cd1—O4—C29	−46.1 (6)	O1—C13—C14—C9	−178.7 (5)
O3—Cd1—O4—C29	−1.1 (5)	C10—C9—C14—O2	178.1 (6)
O5—Cd1—O4—C29	71.2 (5)	C8—C9—C14—O2	−4.1 (9)
O10—Cd1—O4—C29	−79.5 (5)	C10—C9—C14—C13	−1.9 (9)
C2—C3—C4—C5	0.9 (14)	C8—C9—C14—C13	175.9 (6)
O7—N3—O5—Cd1	−178.1 (7)	C22—C17—C18—C19	−0.1 (17)
O6—N3—O5—Cd1	0.2 (7)	C16—C17—C18—C19	179.2 (9)
O2—Cd1—O5—N3	71.9 (5)	C17—C18—C19—C20	−2.3 (17)
O4—Cd1—O5—N3	173.6 (5)	C18—C19—C20—C21	3.3 (16)
O6—Cd1—O5—N3	−0.2 (4)	C18—C19—C20—N2	178.1 (9)
O9—Cd1—O5—N3	−54.1 (5)	C23—N2—C20—C21	20.2 (15)
O3—Cd1—O5—N3	−117.6 (5)	C23—N2—C20—C19	−154.3 (9)
O10—Cd1—O5—N3	−105.0 (5)	C19—C20—C21—C22	−1.9 (16)
C3—C4—C5—C6	−0.5 (12)	N2—C20—C21—C22	−176.5 (9)
C3—C4—C5—N1	−179.1 (7)	C18—C17—C22—C21	1.5 (17)
C8—N1—C5—C4	−173.1 (7)	C16—C17—C22—C21	−177.8 (9)
C8—N1—C5—C6	8.2 (10)	C20—C21—C22—C17	−0.5 (17)
O7—N3—O6—Cd1	178.0 (6)	C20—N2—C23—C24	178.5 (8)
O5—N3—O6—Cd1	−0.3 (8)	N2—C23—C24—C29	1.8 (12)
O2—Cd1—O6—N3	−134.3 (4)	N2—C23—C24—C25	−176.7 (7)
O4—Cd1—O6—N3	−8.9 (6)	C29—C24—C25—C26	−1.5 (12)
O9—Cd1—O6—N3	131.4 (5)	C23—C24—C25—C26	177.1 (8)
O3—Cd1—O6—N3	58.6 (5)	C24—C25—C26—C27	1.9 (14)
O5—Cd1—O6—N3	0.2 (4)	C25—C26—C27—C28	−1.2 (13)
O10—Cd1—O6—N3	141.8 (4)	C30—O3—C28—C27	8.4 (11)
C4—C5—C6—C7	−2.2 (11)	Cd1—O3—C28—C27	177.3 (6)
N1—C5—C6—C7	176.4 (6)	C30—O3—C28—C29	−172.4 (6)
C3—C2—C7—C6	−4.7 (16)	Cd1—O3—C28—C29	−3.6 (7)
C1—C2—C7—C6	−177.7 (8)	C26—C27—C28—O3	179.2 (7)
C5—C6—C7—C2	5.0 (13)	C26—C27—C28—C29	0.1 (11)
C5—N1—C8—C9	−172.6 (6)	Cd1—O4—C29—C24	−178.6 (5)
O8—N4—O9—Cd1	−179.7 (7)	Cd1—O4—C29—C28	−0.5 (8)
O10—N4—O9—Cd1	−2.2 (8)	C23—C24—C29—O4	0.1 (10)
O2—Cd1—O9—N4	67.4 (4)	C25—C24—C29—O4	178.6 (7)
O4—Cd1—O9—N4	−44.2 (5)	C23—C24—C29—C28	−178.1 (6)
O6—Cd1—O9—N4	170.9 (5)	C25—C24—C29—C28	0.4 (10)
O3—Cd1—O9—N4	−87.2 (4)	O3—C28—C29—O4	2.9 (9)
O5—Cd1—O9—N4	−148.9 (4)	C27—C28—C29—O4	−177.9 (6)
O10—Cd1—O9—N4	1.2 (4)	O3—C28—C29—C24	−178.9 (6)
N1—C8—C9—C10	178.8 (6)	C27—C28—C29—C24	0.3 (10)

### Hydrogen-bond geometry (Å, °)

**Table:**

*D*—H···*A*	*D*—H	H···*A*	*D*···*A*	*D*—H···*A*
N1—H1D···O2	0.86	1.94	2.616 (6)	135
N2—H2A···O4	0.86	1.90	2.577 (7)	135

## References

[bb1] Bruker (2006). *APEX2* and *SAINT* Bruker AXS Inc., Madison, Wisconsin, USA.

[bb2] Dominiak, P. M., Grech, E., Barr, G., Teat, S., Mallinson, P. & Woźniak, K. (2003). *Chem. Eur. J.* **9**, 963–970. 10.1002/chem.200390118 12584712

[bb3] Elmali, A., Elerman, Y., Zeyrek, C. T. & Svobod, I. (2003). *Z. Naturforsch. Teil B*, **58**, 433–437.

[bb4] Filarowski, A., Koll, A., Glowiak, T., Majewski, E. & Dziembowska, T. (1998). *Ber. Bunsenges. Phys. Chem.* **102**, 393–402.

[bb5] Müller, R. M., Robson, R. & Separovic, S. (2001). *Angew. Chem. Int. Ed.* **40**, 4385–4386. 10.1002/1521-3773(20011203)40:23<4385::aid-anie4385>3.0.co;2-t 12404422

[bb6] Novitchi, G., Costes, J. P., Tuchagues, J. P., Vendierb, L. & Wernsdorfer, W. (2008). *New J. Chem.* **32**, 197–200.

[bb7] Schiff, H. (1864). *Justus Liebigs Ann. Chem.* **131**, 118–119.

[bb8] Sheldrick, G. M. (1996). *SADABS* University of Göttingen, Germany.

[bb9] Sheldrick, G. M. (2008). *Acta Cryst.* A**64**, 112–122. 10.1107/S0108767307043930 18156677

[bb10] West, B. O. (1960). *J. Chem. Soc.* pp. 4944–4947.

[bb11] Woźniak, K., He, H., Klinowski, J., Jones, W., Dziembowska, T. & Grech, E. (1995). *J. Chem. Soc. Faraday Trans.* **91**, 77–85.

[bb12] Yu, Y. Y., Zhao, G. L. & Wen, Y. H. (2007). *Chin. J. Struct. Chem.* **26**, 1395–1402.

[bb13] Zhao, G.-L., Shi, X. & Ng, S. W. (2007). *Acta Cryst.* E**63**, m267–m268.

[bb14] Zhou, Y.-H. & Zhao, G.-L. (2007). *Acta Cryst.* E**63**, m43–m44.

[bb15] Zhou, M. D., Zhao, J., Li, J., Yue, S., Bao, C. N., Mink, J., Zang, S. L. & Kühn, F. E. (2007). *Chem. Eur. J.* **13**, 158–166. 10.1002/chem.200600863 17066496

